# Supplementary material for: Gene regulatory network inference from sparsely sampled noisy data
Source: Nat Commun. 2020 Jul 13;11:3493. doi: 10.1038/s41467-020-17217-1 (PMC7359369; doi:10.1038/s41467-020-17217-1)
Supplement: Supplementary file 1 — Supplementary Information [file 41467_2020_17217_MOESM1_ESM.pdf]

Supplementary information for the article

"Gene regulatory network inference from sparsely sampled noisy data"

Gonçalves et al.

2020

Supplementary notes

Supplementary note 1: Solvability of the GPDM stochastic differential equation

Consider the stochastic differential equation on $t \in [0, T]$ for some fixed T,

$$\mathrm{d}\mathbf{x}_t = \mathbf{f}(\mathbf{u}_t, \mathbf{x}_t, \omega)\mathrm{d}t + \mathrm{d}\mathbf{w}_t, \qquad \mathbf{x}(0) = \mathbf{x}_0, \tag{1}$$

where ω is an element of the sample space Ω. The initial state $\mathbf{x}_0$ is assumed to be normally distributed, $\mathbf{x}_0 \sim \mathcal{N}(\mathbf{m}, \mathbf{P}_0)$ for some covariance matrix $\mathbf{P}_0$, and $\mathbf{u}_t$ is a smooth deterministic input function. The process noise $\mathbf{w}_t$ is an n-dimensional Brownian motion with diagonal covariance matrix $\mathbf{Q} = \mathrm{diag}(q_1, ..., q_n)$. Each component in $\mathbf{f} = [f_1, ..., f_n]^\top$, $f_i = f_i(\mathbf{u}, \mathbf{x}, \omega)$ conditioned on a trajectory $\mathbf{x}$ is modelled as a Gaussian process. For simplicity, we assume first that each f_i is centred (see Remark 2) and has covariance function k_i depending on the input $\mathbf{u}$ and the state $\mathbf{x}$, that is, $\mathbb{E} f_i(\mathbf{u}, \mathbf{x}, \omega) = 0$ and $\mathbb{E} f_i(\mathbf{u}, \mathbf{x}, \omega) f_i(\mathbf{v}, \mathbf{z}, \omega) = k_i(\mathbf{u}, \mathbf{v}, \mathbf{x}, \mathbf{z})$.

Remark 1. *By Mercer's theorem, each covariance k can be represented as*

$$k(\mathbf{u}, \mathbf{v}, \mathbf{x}, \mathbf{z}) = \sum_{k=1}^{\infty} \lambda_k^2 \phi_k(\mathbf{u}, \mathbf{x}) \phi_k(\mathbf{v}, \mathbf{z})$$

with some basis functions $\{\phi_k\}$. The Gaussian $\mathbf{f}$ with this covariance k can be modelled by

$$\mathbf{f}(\mathbf{u}, \mathbf{x}) = \sum_{k=1}^{\infty} \phi_k(\mathbf{u}, \mathbf{x}) \xi_k,$$

where $\xi_k \sim \mathcal{N}(0, \lambda_k^2)$ are mutually independent. From this it is clear that for given $\mathbf{x}$, $\mathbf{f}(\mathbf{u}, \mathbf{x})$ is Gaussian, whereas for random $\mathbf{x}$, it is usually not.

Throughout the article we make the following assumption on the covariances k_i.

Assumption 1. *For every $i = 1, \ldots, n$, there exists a constant L_i such that*

$$|k_i(\mathbf{u}_t, \mathbf{u}_t, \mathbf{x}, \mathbf{x}) - k_i(\mathbf{u}_t, \mathbf{u}_t, \mathbf{x}, \mathbf{z})| \leq L_i |\mathbf{x} - \mathbf{z}|^2$$

uniformly in $t \in [0, T]$.

Before stating and proving existence and uniqueness result for (1) we need one technical lemma.

Lemma 1. *Suppose that Assumption 1 holds and let $\mathbf{x}, \mathbf{z} \in \mathbb{R}^n$ be arbitrary. Then for any $p \geq 1$ there exists a constant C depending on p and the numbers $L_1, \ldots L_n$ such that*

$$\mathbb{E}|\mathbf{f}(\mathbf{u}_t, \mathbf{x}, \omega) - f(\mathbf{u}_t, \mathbf{z}, \omega)|^p \leq C|\mathbf{x} - \mathbf{z}|^p.$$

Proof. Since $\mathbf{f}$ is a Gaussian vector, it suffices to prove the claim only for $p = 2$. Furthermore, by triangle inequality, it suffices to prove that for each component f_i we have

$$\mathbb{E}|f_i(\mathbf{u}_t, \mathbf{x}, \omega) - f_i(\mathbf{u}_t, \mathbf{z}, \omega)|^2 \leq C|\mathbf{x} - \mathbf{z}|^2.$$

Now

$$\mathbb{E}|f_i(\mathbf{u}_t, \mathbf{x}, \omega) - f_i(\mathbf{u}_t, \mathbf{z}, \omega)|^2 = k_i(\mathbf{u}_t, \mathbf{u}_t, \mathbf{x}, \mathbf{x}) + k_i(\mathbf{u}_t, \mathbf{u}_t, \mathbf{z}, \mathbf{z}) - 2k_i(\mathbf{u}_t, \mathbf{u}_t, \mathbf{x}, \mathbf{z}),$$

and Assumption 1 implies

$$\mathbb{E}|f_i(\mathbf{u}_t, \mathbf{x}, \omega) - f_i(\mathbf{u}_t, \mathbf{z}, \omega)|^2 \leq 2L_i|\mathbf{x} - \mathbf{z}|^2$$

which concludes the proof. □

Corollary 1. *Suppose that Assumption 1 holds and let* $\mathbf{x}, \mathbf{z} \in \mathbb{R}^n$ *be random variables. Then for any* $p \geq 1$ *there exists a constant* C *depending on* p *and the numbers* $L_1, \ldots L_n$ *such that*

$$\mathbb{E}\left(|\mathbf{f}(\mathbf{u}_t, \mathbf{x}, \omega) - f(\mathbf{u}_t, \mathbf{z}, \omega)|^p \,|\, \mathbf{x}, \mathbf{z}\right) \leq C|\mathbf{x} - \mathbf{z}|^p.$$

Proof. The claim follows from Lemma 1 together with the fact that f_i conditioned on $\mathbf{x}$ and $\mathbf{z}$ is Gaussian with covariance k_i. □

The following existence and uniqueness result for the stochastic differential equation (1) justifies the use of the continuous-time GPDM.

Theorem 1. *Suppose that Assumption 1 is satisfied. Then* (1) *admits a unique solution* x.

Proof. We use Picard iteration and define

$$\mathbf{x}_t^0 = \mathbf{x}_0,$$

and for $j \geq 1$ we set

$$\mathbf{x}_t^j = \mathbf{x}_0 + \int_0^t \mathbf{f}(\mathbf{u}_s, \mathbf{x}_s^{j-1}, \omega)\mathrm{d}s + \mathbf{w}_t - \mathbf{w}_0.$$

Then

$$\mathbf{x}_t^j - \mathbf{x}_t^{j-1} = \int_0^t \mathbf{f}(\mathbf{u}_s, \mathbf{x}_s^{j-1}, \omega) - \mathbf{f}(\mathbf{u}_s, \mathbf{x}_s^{j-2}, \omega)\mathrm{d}s$$

and

$$|\mathbf{x}_t^j - \mathbf{x}_t^{j-1}| \leq \int_0^t |\mathbf{f}(\mathbf{u}_s, \mathbf{x}_s^{j-1}, \omega) - \mathbf{f}(\mathbf{u}_s, \mathbf{x}_s^{j-2}, \omega)|\mathrm{d}s. \tag{2}$$

Taking expectation, conditioning, and using Corollary 1 then gives

$$\mathbb{E}|\mathbf{x}_t^j - \mathbf{x}_t^{j-1}| \leq C\int_0^t \mathbb{E}|\mathbf{x}_s^{j-1} - \mathbf{x}_s^{j-2}|\mathrm{d}s. \tag{3}$$

We now claim that

$$\mathbb{E}|\mathbf{x}_t^j - \mathbf{x}_t^{j-1}| \le \frac{C_1 C^j t^j}{j!} + \frac{C_2 C^{j-1} t^{j-1}}{(j-1)!}.$$

This follows by induction. For $j = 1$ we have

$$|\mathbf{x}_t^1 - \mathbf{x}_0| = \left| \int_0^t \mathbf{f}(\mathbf{u}_s, \mathbf{x}_0) \mathrm{d}s + \mathbf{w}_t - \mathbf{w}_0 \right| \le \sup_{s\in[0,T]} |\mathbf{f}(\mathbf{u}_s, \mathbf{x}_0)| t + \sup_{s\in[0,T]} |\mathbf{w}_t|$$

which proves the claim for $j = 1$ as the supremum of Gaussian process $\mathbf{f}$ and the supremum of $\mathbf{w}_t$ have all moments finite. Suppose

$$\mathbb{E}|\mathbf{x}_s^j - \mathbf{x}_s^{j-1}| \le \frac{C_1 C^j s^j}{j!} + \frac{C_2 C^{j-1} s^{j-1}}{(j-1)!}.$$

Then (3) implies

$$\mathbb{E}|\mathbf{x}_t^{j+1} - \mathbf{x}_t^j| \le \int_0^t \frac{C_1 C^{j+1} s^j}{j!} + \frac{C_2 C^j s^j}{(j-1)!} \mathrm{d}s = \frac{C_1 C^{j+1} t^{j+1}}{(j+1)!} + \frac{C_2 C^j t^j}{j!}.$$

In particular, this gives

$$\sup_{t\in[0,T]} \mathbb{E}|\mathbf{x}_t^{j+1} - \mathbf{x}_t^j| \le \frac{C_1 C^{j+1} T^{j+1}}{(j+1)!} + \frac{C_2 C^j T^j}{j!} \to 0$$

and

$$\sum_{j=0}^{\infty} \sup_{t\in[0,T]} \mathbb{E}|\mathbf{x}_t^{j+1} - \mathbf{x}_t^j| < \infty.$$

On the other hand, from (2) we get

$$\sup_{t\in[0,T]} |\mathbf{x}_t^j - \mathbf{x}_t^{j-1}| \le \int_0^T |\mathbf{f}(\mathbf{u}_s, \mathbf{x}_s^{j-1}, \omega) - \mathbf{f}(\mathbf{u}_s, \mathbf{x}_s^{j-2}, \omega)| \mathrm{d}s.$$

Consequently, taking expectation gives

$$\mathbb{E}\left[\sup_{t\in[0,T]} |\mathbf{x}_t^j - \mathbf{x}_t^{j-1}|\right] \le CT \sup_{t\in[0,T]} \mathbb{E}|\mathbf{x}_t^{j-1} - \mathbf{x}_t^{j-2}|,$$

and thus we also have

$$\sum_{j=0}^{\infty} \mathbb{E}\left[\sup_{t\in[0,T]} |\mathbf{x}_t^{j+1} - \mathbf{x}_t^j|\right] < \infty.$$

This implies that

$$\sum_{j=0}^{k} (\mathbf{x}_t^{j+1} - \mathbf{x}_t^j) = \mathbf{x}_t^{k+1} - \mathbf{x}_0$$

converges uniformly to an integrable random variable. Finally, since $\mathbf{f}(\mathbf{u}_s, \mathbf{x}, \omega)$ is continuous in $\mathbf{x}$ by Gaussianity and Lemma 1, we observe that the limit $\mathbf{x} = \lim_{j\to\infty} \mathbf{x}^j$ satisfies (1). □

Remark 2. *We stress that while we assumed the Gaussian process* $\mathbf{f}$ *to be centred for the sake of simplicity, the extension to a non-centred case is rather straightforward. Indeed, if for each component* f_i *the mean function* $\mathbb{E}f_i(\mathbf{u}_t, \mathbf{x}, \omega) = m_i(\mathbf{u}_t, \mathbf{x})$ *is Lipschitz continuous with respect to* $\mathbf{x}$ *uniformly in* t*, i.e.*

$$|m_i(\mathbf{u}_t, \mathbf{x}) - m_i(\mathbf{u}_t, \mathbf{z})| \leq L_i|\mathbf{x} - \mathbf{z}|,$$

then the existence and uniqueness follows from the above proof by centering $\mathbf{f}$ *first. We leave the details to the reader.*

The following result studies the basic properties of the solution.

Theorem 2. *Suppose that Assumption 1 holds. Then the solution* $\mathbf{x}$ *to* (1) *is Hölder continuous of any order* $\gamma < \frac{1}{2}$. *Furthermore,* $\sup_{t\in[0,T]} |\mathbf{x}_t|$ *has all the moments finite.*

Proof. Clearly, each $\mathbf{x}^j$ in the proof of Theorem 1 is continuous. Consequently, the solution $\mathbf{x}$ is continuous as a uniform limit of continuous trajectories. The Hölder continuity then follows from (1) and the Hölder continuity of the Brownian motion $\mathbf{w}$. Indeed, since $\mathbf{f}(\mathbf{u}_s, \mathbf{x}, \omega)$ is continuous in $\mathbf{x}$ and $\mathbf{x}$ is bounded as a continuous function on a bounded interval $[0, T]$, it follows that $\mathbf{f}(\mathbf{u}_s, \mathbf{x}_s, \omega)$ is also bounded. Finally, the existence of all moments follow from the fact that $\mathbf{f}(\mathbf{u}_s, \mathbf{x}_s, \omega)$ has all the moments finite as well as $\sup_{t\in[0,T]} |\mathbf{w}_t|$ has all the moments finite. $\square$

Supplementary note 2: Convergence of the Euler discretisation

The second part of the theoretical considerations is concerned with convergence of the Euler discretised trajectory that is defined on a partition $\pi^M = \{0 = \tau_0 < \tau_1 < \ldots < \tau_M = T\}$ of the compact interval of interest $[0, T]$ by

$$\mathbf{X}^M_{\tau_k} = \mathbf{X}^M_{\tau_{k-1}} + \delta\tau_k \mathbf{f}(\mathbf{u}_{\tau_{k-1}}, \mathbf{X}^M_{\tau_{k-1}}, \omega) + \mathbf{w}_{\tau_k} - \mathbf{w}_{\tau_{k-1}} \tag{4}$$

where $\delta\tau_k := \tau_k - \tau_{k-1}$, and $k = 1, ..., M$. Later, we will obtain a probability distribution for the discrete trajectory $\mathbf{X} = [\mathbf{X}_{\tau_0}, \mathbf{X}_{\tau_1}, ..., \mathbf{X}_{\tau_M}]$, but first we show the pointwise (in ω) convergence to the continuous solution of (1) as the temporal discretisation is refined.

We study the continuous version defined for $t \in [\tau_{k-1}, \tau_k]$ by

$$\overline{\mathbf{X}}^M_t = \overline{\mathbf{X}}^M_{\tau_{k-1}} + (t - \tau_{k-1})\mathbf{f}(\mathbf{u}_{\tau_{k-1}}, \overline{\mathbf{X}}^M_{\tau_{k-1}}, \omega) + \mathbf{w}_t - \mathbf{w}_{\tau_{k-1}}. \tag{5}$$

Note that $\mathbf{X}^M_{\tau_k} = \overline{\mathbf{X}}^M_{\tau_k}$ for all k.

Theorem 3. *Suppose that Assumption 1 holds and consider arbitrary discretisation partition π^M such that $\sup_M |\pi^M| M < \infty$, where $|\pi^M| = \max_k(\tau_k - \tau_{k-1})$. Then for any $p \geq 1$*

$$\mathbb{E}\left[\sup_{t\in[0,T]} |\mathbf{x}_t - \overline{\mathbf{X}}_t^M|\right]^p \leq C|\pi^M|^p.$$

Moreover, for any $\epsilon > 0$ we have

$$\sup_{t\in[0,T]} |\mathbf{x}_t - \overline{\mathbf{X}}_t^M| \leq C|\pi^M|^{1-\epsilon}.$$

almost surely.

Proof. Let $t \in [\tau_{k-1}, \tau_k]$ and denote

$$z_k = \left\| \sup_{t\in[\tau_{k-1},\tau_k]} |\mathbf{x}_t - \overline{\mathbf{X}}_t^M| \right\|_p,$$

where $\|\cdot\|_p$ denotes the p-norm. Now

$$\mathbf{x}_t - \overline{\mathbf{X}}_t^M = \mathbf{x}_{\tau_{k-1}} - \overline{\mathbf{X}}_{\tau_{k-1}}^M + \int_{\tau_{k-1}}^t \mathbf{f}(\mathbf{u}_s, \mathbf{x}_s, \omega) - \mathbf{f}(\mathbf{u}_{\tau_{k-1}}, \overline{\mathbf{X}}_{\tau_{k-1}}^M, \omega)\mathrm{d}s.$$

As in the proof of Theorem 1, this implies

$$z_k \leq z_{k-1} + \int_{\tau_{k-1}}^{\tau_k} \left\| \mathbf{f}(\mathbf{u}_s, \mathbf{x}_s, \omega) - \mathbf{f}(\mathbf{u}_{\tau_{k-1}}, \overline{\mathbf{X}}_{\tau_{k-1}}^M, \omega) \right\|_p \mathrm{d}s \leq z_{k-1} + Cz_k|\pi^M|.$$

Let now M be large enough such that $C|\pi^M| < 1$. We get

$$(1 - C|\pi^M|)z_k \leq z_{k-1}$$

or equivalently

$$z_k \leq \frac{1}{1 - C|\pi^M|} z_{k-1}.$$

Iterating then gives

$$z_k \leq \left(\frac{1}{1 - C|\pi^M|}\right)^k z_1 = \left(1 + \frac{C}{|\pi^M|^{-1} - C}\right)^k z_1.$$

Note next that

$$\left(1 + \frac{C}{|\pi^M|^{-1} - C}\right)^k \leq \left(1 + \frac{\tilde{C}}{|\pi^M|^{-1}}\right)^M = \left(1 + \frac{\tilde{C}}{|\pi^M|^{-1}}\right)^{|\pi^M|^{-1}|\pi^M|M}$$

for some other constant $\tilde{C}$. Since

$$\left(1 + \frac{\tilde{C}}{|\pi^M|^{-1}}\right)^{|\pi^M|^{-1}} \to e^{\tilde{C}}$$

as $M \to \infty$ and $|\pi^M|M$ is bounded by assumption, it follows that

$$z_k \leq C z_1$$

for some unimportant constant C. But now

$$|\mathbf{x}_t - \overline{\mathbf{X}}_t^M| \leq \int_0^{\tau_1} |\mathbf{f}(\mathbf{u}_s, \mathbf{x}_s, \omega) - \mathbf{f}(\mathbf{u}_{\tau_{k-1}}, \overline{\mathbf{X}}^M_{\tau_{k-1}}, \omega)|\mathrm{d}s \leq 2 \sup_{s\in[0,T]} |\mathbf{f}(\mathbf{u}_s, \mathbf{x}_s, \omega)||\pi^M|$$

for $t \in [0, \tau_1]$ from which it follows that $z_1 \leq C|\pi^M|$ proving the first claim. Finally, the second claim is a direct consequence of the Borel-Cantelli lemma.

□

Supplementary note 3: Probability distribution of the discretised trajectory

For the discrete-time GPDM, the probability distribution of the discretised trajectory is computed in [1, Appendix A], and for the most parts the derivation here is similar.

Like in the main text, only one discretisation level is considered hereinafter, and the discretisation index is dropped, that is, $\mathbf{X} = \mathbf{X}^M$. For notational simplicity, we first assume that the Gaussian process $\mathbf{f}$ is centred, that is, $m_i(\mathbf{u}, \mathbf{x}) = 0$. It holds that

$$p(\mathbf{X}|\theta) = \int p(\mathbf{X}|f, \theta) p(\mathbf{f}|\theta) \mathrm{d}\mathbf{f}. \tag{6}$$

For given $\mathbf{f}$, the trajectory $\mathbf{X}$ is a Markov process, and therefore its distribution satisfies

$$p(\mathbf{X}|\mathbf{f}, \theta) = p(\mathbf{X}_{\tau_0}|\theta) \prod_{k=1}^{M} p(\mathbf{X}_{\tau_k}|\mathbf{X}_{\tau_{k-1}}, \mathbf{f}, \theta)$$

where

$$p(\mathbf{X}_{\tau_k}|\mathbf{X}_{\tau_{k-1}}, \mathbf{f}, \theta) = \frac{1}{(2\pi\delta\tau_k)^{n/2}|\mathbf{Q}|^{1/2}} \exp\left(-\frac{1}{2\delta\tau_k} \left|\mathbf{X}_{\tau_k} - \mathbf{X}_{\tau_{k-1}} - \delta\tau_k \mathbf{f}(\mathbf{X}_{\tau_{k-1}})\right|^2_{\mathbf{Q}^{-1}}\right).$$

Let us introduce notation $\overline{\mathbf{X}} := [\mathbf{X}_{\tau_1}, \ldots, \mathbf{X}_{\tau_M}]^\top$ and $\underline{\mathbf{X}} := [\mathbf{X}_{\tau_0}, \ldots, \mathbf{X}_{\tau_{M-1}}]^\top$. Same notation is also used for the different dimensions of the trajectory.

Then it holds that

$$
\begin{aligned}
&p(\mathbf{X}|\mathbf{f},\theta) \\
&= \frac{p(\mathbf{X}_{\tau_0}|\theta)}{(2\pi)^{Mn/2}|\mathbf{Q}|^{M/2}} \prod_{k=1}^{M} \frac{1}{\delta\tau_k^{n/2}} \exp\left(-\frac{1}{2\delta\tau_k}\left|\mathbf{X}_{\tau_k} - \mathbf{X}_{\tau_{k-1}} - \delta\tau_k \mathbf{f}(\mathbf{X}_{\tau_{k-1}})\right|^2_{\mathbf{Q}^{-1}}\right) \\
&= \frac{p(\mathbf{X}_{\tau_0}|\theta)}{(2\pi)^{Mn/2}|\mathbf{Q}|^{M/2}|\Delta\tau|^{n/2}} \exp\left(-\sum_{k=1}^{M} \frac{1}{2\delta\tau_k}\left|\mathbf{X}_{\tau_k} - \mathbf{X}_{\tau_{k-1}} - \delta\tau_k \mathbf{f}(\mathbf{X}_{\tau_{k-1}})\right|^2_{\mathbf{Q}^{-1}}\right) \\
&= \frac{p(\mathbf{X}_{\tau_0}|\theta)}{(2\pi)^{Mn/2}|\mathbf{Q}|^{M/2}|\Delta\tau|^{n/2}} \prod_{i=1}^{n} \exp\left(-\frac{1}{2q_i}\left|\overline{\mathbf{X}}_i - \underline{\mathbf{X}}_i - \Delta\tau f_i(\underline{\mathbf{X}})\right|^2_{\Delta\tau^{-1}}\right)
\end{aligned}
$$

where $\Delta\tau$ is a diagonal matrix whose element (k,k) is $\delta\tau_k$, and $f_i(\underline{\mathbf{X}}) = \left[f_i(\mathbf{X}_{\tau_0}), \ldots, f_i(\mathbf{X}_{\tau_{M-1}})\right]^\top$.

Now $p(\mathbf{X}|\mathbf{f},\theta)$ in the integral (6) depends only on the values of $\mathbf{f}$ at points $\underline{\mathbf{X}}$. By definition of a Gaussian process, the integral can equivalently be computed over a collection of finite-dimensional, normally distributed random variables $\mathbf{F} = [\mathbf{F}_1, \ldots, \mathbf{F}_n] \in \mathbb{R}^{M\times n}$ where $\mathbf{F}_i \in \mathbb{R}^M$ has mean zero, and covariance $\mathbf{K}_i(\underline{\mathbf{X}})$ given elementwise by $[\mathbf{K}_i(\underline{\mathbf{X}})]_{j,k} = k_i(\mathbf{X}_{\tau_{j-1}}, \mathbf{X}_{\tau_{k-1}})$. The integral in (6) can be computed analytically,

$$
\begin{aligned}
&\int p(\mathbf{X}|\mathbf{f},\theta)p(\mathbf{f}|\theta)\mathrm{d}\mathbf{f} \\
&= \frac{p(\mathbf{X}_{\tau_0}|\theta)}{(2\pi)^{Mn}|\mathbf{Q}|^{M/2}|\Delta\tau|^{n/2}} \\
&\prod_{i=1}^{n} \int \frac{1}{|\mathbf{K}_i(\underline{\mathbf{X}})|^{1/2}} \exp\left(-\frac{1}{2q_i}\left|\overline{\mathbf{X}}_i - \underline{\mathbf{X}}_i - \Delta\tau\mathbf{F}_i\right|^2_{\Delta\tau^{-1}} - \frac{1}{2}\left|\mathbf{F}_i\right|^2_{\mathbf{K}(\underline{\mathbf{X}})^{-1}}\right)\mathrm{d}\mathbf{F}_i \\
&= \frac{p(\mathbf{X}_{\tau_0}|\theta)}{(2\pi)^{Mn/2}|\mathbf{Q}|^{M/2}|\Delta\tau|^{n/2}} \prod_{i=1}^{n} \frac{1}{|\mathbf{K}_i(\underline{\mathbf{X}})|^{1/2}\left|\frac{\Delta\tau}{q_i} + \mathbf{K}_i(\underline{\mathbf{X}})^{-1}\right|^{1/2}} \\
&\exp\left(-\frac{1}{2q_i}\left|\overline{\mathbf{X}}_i - \underline{\mathbf{X}}_i\right|^2_{\Delta\tau^{-1}} + \frac{1}{2q_i^2}(\overline{\mathbf{X}}_i - \underline{\mathbf{X}}_i)^\top\left(\frac{\Delta\tau}{q_i} + \mathbf{K}_i(\underline{\mathbf{X}})\right)^{-1}(\overline{\mathbf{X}}_i - \underline{\mathbf{X}}_i)\right).
\end{aligned}
$$

Applying the Woodbury identity to the exponent gives

$$
(q_i\Delta\tau)^{-1} - \frac{1}{q_i}\Big(\Delta\tau(q_i\Delta\tau)^{-1}\Delta\tau + \mathbf{K}_i(\underline{\mathbf{X}})^{-1}\Big)\frac{1}{q_i} = \left(\Delta\tau\mathbf{K}_i(\underline{\mathbf{X}})\Delta\tau + q_i\Delta\tau\right)^{-1},
$$

and the determinant lemma gives (recall $\mathbf{Q}$ is a diagonal matrix with q_i's on

the diagonal)

$$
\begin{aligned}
&|\mathbf{Q}|^{M/2}|\Delta\tau|^{n/2}\prod_{i=1}^{n}|\mathbf{K}_i(\underline{\mathbf{X}})|^{1/2}\left|\frac{\Delta\tau}{q_i}+\mathbf{K}_i(\underline{\mathbf{X}})^{-1}\right|^{1/2}\\
&=\prod_{i=1}^{n}|q_i\Delta\tau||\mathbf{K}_i(\underline{\mathbf{X}})|^{1/2}\left|\Delta\tau(q_i\Delta\tau)^{-1}\Delta\tau+\mathbf{K}_i(\underline{\mathbf{X}})^{-1}\right|\\
&=\prod_{i=1}^{n}\left|\Delta\tau\mathbf{K}_i(\underline{\mathbf{X}})\Delta\tau+q_i\Delta\tau\right|^{1/2}.
\end{aligned}
$$

Finally, the desired probability distribution is

$$
\begin{aligned}
p(\mathbf{X}|\theta) =&\frac{p(\mathbf{X}_{\tau_0}|\theta)}{(2\pi)^{Mn/2}}\prod_{i=1}^{n}\frac{1}{|\Delta\tau\mathbf{K}_i(\underline{\mathbf{X}})\Delta\tau+q_i\Delta\tau|^{1/2}} &(7)\\
&\cdot\exp\left(-\frac{1}{2}(\overline{\mathbf{X}}_i-\underline{\mathbf{X}}_i)^\top\left(\Delta\tau\mathbf{K}_i(\underline{\mathbf{X}})\Delta\tau+q_i\Delta\tau\right)^{-1}(\overline{\mathbf{X}}_i-\underline{\mathbf{X}}_i)\right)\\
=:&\frac{p(\mathbf{X}_{\tau_0}|\theta)}{(2\pi)^{Mn/2}}\prod_{i=1}^{n}P_i(\mathbf{S}_i,\mathbf{H}_i,\gamma_i,q_i,a_i,b_i,\mathbf{X}) &(8)
\end{aligned}
$$

To take into account the nonzero mean $m_i(\mathbf{x}) = b_i - a_i x_i$ of the Gaussian process f_i that is used in the method, the differences $\overline{\mathbf{X}}_i - \underline{\mathbf{X}}_i$ are replaced by $\overline{\mathbf{X}}_i - \underline{\mathbf{X}}_i - \Delta\tau(b_i - a_i\underline{\mathbf{X}}_i)$. Variables $\mathbf{S}_i$, $\mathbf{H}_i$, and γ_i in P_i are parameters of the covariance function k_i.

Note that above it was implicitly assumed that the covariance $\mathbf{K}_i(\underline{\mathbf{X}})$ is positive definite. This assumption is only violated if $\mathbf{X}_{\tau_j} = \mathbf{X}_{\tau_k}$ for some $j \neq k$ or if the covariance function k_i is degenerate. In this case the integral should be computed over a lower-dimensional variable $\mathbf{F}_i$, but the end result would not change.

Note also that (7) corresponds to the finite dimensional distribution of the continuous Euler scheme (5) evaluated at discretisation points. Since (5) converges strongly to the solution $\mathbf{x}$ of (1), the finite dimensional distributions converge as well. This means that (7) is a finite dimensional approximation of the distribution of $\mathbf{x}$.

In the derivation of the probability distribution $p(\mathbf{X}|\theta)$, an integral of the exponential function with a quadratic exponent was computed. Here it is shown how such integral is computed analytically.

Consider the integral

$$\int_{\mathbb{R}^N}\exp(-J(\mathbf{x}))\mathrm{d}\mathbf{x}$$

where

$$J(\mathbf{x}) = \langle\mathbf{x},\mathbf{A}\mathbf{x}\rangle + \langle\mathbf{b},\mathbf{x}\rangle + c,$$

and $\mathbf{A}$ is symmetric and positive definite. Now J can be written as

$$J(\mathbf{x}) = J_{\min} + \langle\mathbf{x}-\mathbf{x}_{\min},\mathbf{A}(\mathbf{x}-\mathbf{x}_{\min})\rangle$$

where $J_{\min} = \min_{\mathbf{x}} J(\mathbf{x})$ and $\mathbf{x}_{\min}$ is the (unique) vector attaining this minimum. Then

$$\begin{aligned}\int_{\mathbb{R}^N} \exp(-J(\mathbf{x}))\mathrm{d}\mathbf{x} &= \exp(-J_{\min}) \int_{\mathbb{R}^N} \exp\big(- \langle \mathbf{x} - \mathbf{x}_{\min}, \mathbf{A}(\mathbf{x} - \mathbf{x}_{\min})\rangle \big)\mathrm{d}\mathbf{x} \\ &= \exp(-J_{\min}) \int_{\mathbb{R}^N} \exp\big(- \langle \mathbf{x}, \mathbf{A}\mathbf{x}\rangle \big)\mathrm{d}\mathbf{x} \\ &= \frac{\pi^{N/2}}{|\mathbf{A}|^{1/2}} \exp(-J_{\min}).\end{aligned}$$

Finally, the minimum is obtained by straightforward differentiation, and it is

$$J_{\min} = c - \frac{1}{4}\left\langle \mathbf{b}, \mathbf{A}^{-1}\mathbf{b} \right\rangle.$$

In the derivation of $p(\mathbf{X}|\theta)$ above, this is applied so that

$$\begin{cases} \mathbf{A} = \frac{1}{2q_i}\Delta\tau + \frac{1}{2}\mathbf{K}_i(\underline{\mathbf{X}})^{-1}, \\ \mathbf{b} = -\frac{1}{q_i}(\overline{\mathbf{X}}_i - \underline{\mathbf{X}}_i), \\ c = \frac{1}{2q_i}\left|\overline{\mathbf{X}}_i - \underline{\mathbf{X}}_i\right|^2_{\Delta\tau^{-1}}. \end{cases}$$

Supplementary note 4: Network inference algorithm

As mentioned in the main text, the covariance function for each component f_i of $\mathbf{f}$ in (1) is modelled as a Gaussian process with the squared exponential covariance function

$$k_i(\mathbf{u}, \mathbf{v}, \mathbf{x}, \mathbf{z}) = \gamma_i \exp\left(- \sum_{j=1}^{n} \beta_{i,j}(x_j - z_j)^2 - \sum_{j=1}^{m} \beta_{i,n+j}(u_j - v_j)^2 \right)$$

where n is the system dimension and m is the dimension of the input $\mathbf{u}$ in (1). The method is based on estimating posterior probabilities for $\beta_{i,j}$ for $i = 1, ..., n$ and $j = 1, ..., n+m$ being nonzero. Indeed, if $\beta_{i,j} = 0$, it means that the function f_i does not depend on x_j, that is, gene j is not a regulator of gene i. In a similar way, the method estimates also the target genes of the external inputs u_j for $j = 1, ..., m$. The hyperparameters $\beta_{i,j}$ are given a so-called spike-and-slab prior, meaning that there is a positive probability that $\beta_{i,j} = 0$. One way to treat such random variables in practice is to represent them as a product $\beta_{i,j} = S_{i,j}H_{i,j}$ of an indicator variable $S_{i,j} \in \{0, 1\}$ and a magnitude variable $H_{i,j} \in \mathbb{R}^+$. The indicator variable matrix $\mathbf{S}$ then has the interpretation of the adjacency matrix of the underlying gene regulatory network.

The network inference algorithm is based on MCMC sampling of the probability distribution

$$p(\theta|\mathbf{Y}) \propto \int p(\mathbf{Y}|\mathbf{x}, \theta)p(\mathbf{x}|\theta)p(\theta)\mathrm{d}\mathbf{x}$$

Supplementary table 1: Parameter summary.

Symbol	Explanation
$\beta_{i,j}$	Inverse length scale in the GP covariance, $\beta_{i,j} = S_{i,j} H_{i,j}$
$S_{i,j} \in \{0,1\}$	Indicator variable for $\beta_{i,j}$
$H_{i,j} \in \mathbb{R}^+$	Magnitude variable for $\beta_{i,j}$
γ_i	Scale of the GP covariance
a_i	mRNA degradation rate in the GP mean
b_i	basal transcription rate in the GP mean
$\mathbf{Q} = \mathrm{diag}(q_i)$	incremental covariance of the process noise
$\mathbf{R} = \mathrm{diag}(r_i)$	measurement error variance
$M_{i,\mathrm{ss}}$	variance of $\mathbf{f}$ at steady-state
$M_{i,\mathrm{ko}}$	variance of $\mathbf{f}$ at the ko/kd steady states

where $\theta = \{S_{i,j}, H_{i,j}, \gamma_i, q_i, r_i, a_i, b_i\}_{i=1,...,n,\ j=1,...,n+m}$. Here $p(\mathbf{Y}|\mathbf{x}, \theta)$ corresponds to the measurement model $\mathbf{y}_k = \mathbf{x}(t_k) + \mathbf{v}_k$ where $\mathbf{v}_k \sim \mathcal{N}(0, \mathbf{R})$, and $\mathbf{v}_{k_1} \perp \mathbf{v}_{k_2}$ for $k_1 \neq k_2$. The distribution $p(\mathbf{x}|\theta)$ is approximated by the discretised $p(\mathbf{X}|\theta)$ given in (7), and $p(\theta)$ consists of hyperparameter priors, specified below. The output of the algorithm is the average of the collected samples of network topologies $\mathbf{S}$. The trajectory $\mathbf{X}$ and all other hyperparameters besides $\mathbf{S}$ are integrated out by sampling. The probability distribution $p(\mathbf{X}|\theta)$ is readily factorised in form (8). Each factor P_i only depends on the ith component/row of the vectors/matrices $\mathbf{S}$, $\mathbf{H}$, γ, $\mathbf{q}$, $\mathbf{a} = \{a_1, ..., a_n\}$, and $\mathbf{b} = \{b_1, ..., b_n\}$, and so it is natural to sample them one dimension at a time. However, each factor P_i still depends on the full trajectory $\mathbf{X}$, so the trajectory sampling is done separately. Also, when using the Crank–Nicolson sampling (explained below), the sampling of $\mathbf{q}$ is intertwined with the trajectory sampling, so they are sampled together. This two-phase sampling scheme is described in the following algorithm. Here the algorithm is presented in its basic form. Some ways to make the sampling more efficient are discussed below. We assume that the initial time τ_0 coincides with the time of the first measurement t_0, so that $p(\mathbf{X}_{\tau_0}|\theta) = \mathcal{N}(\mathbf{y}_0, \mathbf{R})$. In the algorithm, this is included in the data fit term $p(\mathbf{Y}|\mathbf{x}, \theta)$.

Algorithm 1. *Denote the lth samples by parenthesised superindex, e.g., $\mathbf{X}^{(l)}$ is the trajectory of the lth sample. The new candidate samples are denoted by a hat. A parameter summary is in Supplementary table 1. Prior probability distributions used in the method are described below.*

Indicator and hyperparameter sampling:

For $i = 1, ..., n$:

- *Sample the ith row of $\mathbf{S}$ by drawing $\hat{j}$ from uniform distribution over $\{1, ..., n\}$. Then*

$$\hat{S}_{i,j} = \begin{cases} S_{i,j}^{(l)}, & \text{if } j \neq \hat{j}, \\ 1 - S_{i,j}^{(l)}, & \text{if } j = \hat{j}. \end{cases}$$

- *Sample* $\mathbf{H}_i = [H_{i,1}, ..., H_{i,n}]$, γ_i, a_i, *and* b_i *using random walk sampling, that is, add small changes to each component, drawn from zero-mean normal distribution. If the candidate sample is negative, take its absolute value.*

- *Accept the new samples with probability*

$$\frac{P_i(\hat{\mathbf{S}}_i, \hat{\mathbf{H}}_i, \hat{\gamma}_i, q_i^{(l)}, \hat{a}_i, \hat{b}_i, \mathbf{X}^{(l)})p(\hat{\mathbf{S}}_i, \hat{\mathbf{H}}_i, \hat{\gamma}_i, q_i^{(l)}, \hat{a}_i, \hat{b}_i)}{P_i(\mathbf{S}_i^{(l)}, \mathbf{H}_i^{(l)}, \gamma_i^{(l)}, q_i^{(l)}, a_i^{(l)}, b_i^{(l)}, \mathbf{X}^{(l)})p(\mathbf{S}_i^{(l)}, \mathbf{H}_i^{(l)}, \gamma_i^{(l)}, q_i^{(l)}, a_i^{(l)}, b_i^{(l)})}$$

where p *is composed of the hyperparameter priors, and the factors* P_i *are defined in* (8)*.*

- *Sample* $\hat{\mathbf{R}}$ *with random walk sampling, with acceptance probability*

$$\frac{p(\hat{\mathbf{R}})|\mathbf{R}^{(l)}|^{(m+1)/2}}{p(\mathbf{R}^{(l)})|\hat{\mathbf{R}}|^{(m+1)/2}} \exp\left(\frac{1}{2}\sum_{j=0}^{m} \left|\mathbf{y}_j - \mathbf{X}^{(l)}\mathbf{C}_j\right|^2_{(\mathbf{R}^{(l)})^{-1}} - \left|\mathbf{y}_j - \mathbf{X}^{(l)}\mathbf{C}_j\right|^2_{\hat{\mathbf{R}}^{-1}}\right)$$

Trajectory sampling:

- *Sample* $\hat{\mathbf{X}}_i = \mathbf{X}_i^{(l)} + \mathbf{B}\mathbf{g}$, *where* $\mathbf{g} \sim \mathcal{N}(0, \varepsilon\mathbf{I})$, $\mathbf{B} = [\mathbf{b}_1, ..., \mathbf{b}_{2m_b}]$, *and*

$$b_j = \begin{cases} \frac{1}{j}\left[\sin\left(\frac{2\pi j\tau_0}{T}\right), ..., \sin\left(\frac{2\pi j\tau_M}{T}\right)\right]^\top, & j = 1, ..., m_b, \\ \frac{1}{j-m_b}\left[\cos\left(\frac{2\pi(j-m_b)\tau_0}{T}\right), ..., \cos\left(\frac{2\pi(j-m_b)\tau_M}{T}\right)\right]^\top, & j = m_b+1, ..., 2m_b, \end{cases}$$

where $m_b = \lfloor M/2 \rfloor$*.*

- *Sample* $\hat{\mathbf{Q}}$ *using the random walk sampling.*

- *Accept* $\hat{\mathbf{X}}$ *and* $\hat{\mathbf{Q}}$ *with probability*

$$\frac{p(\hat{\mathbf{Q}})}{p(\mathbf{Q}^{(l)})} \exp\left(\frac{1}{2}\sum_{j=0}^{m} \left|\mathbf{y}_j - \mathbf{X}^{(l)}\mathbf{C}_j\right|^2_{(\mathbf{R}^{(l+1)})^{-1}} - \left|\mathbf{y}_j - \hat{\mathbf{X}}\mathbf{C}_j\right|^2_{(\mathbf{R}^{(l+1)})^{-1}}\right)$$
$$\times \prod_{i=1}^{n} \frac{P_i(\mathbf{S}_i^{(l+1)}, \mathbf{H}_i^{(l+1)}, \gamma_i^{(l+1)}, \hat{q}_i, a_i^{(l+1)}, b_i^{(l+1)}, \hat{\mathbf{X}})}{P_i(\mathbf{S}_i^{(l+1)}, \mathbf{H}_i^{(l+1)}, \gamma_i^{(l+1)}, q_i^{(l)}, a_i^{(l+1)}, b_i^{(l+1)}, \mathbf{X}^{(l)})}$$

where $\mathbf{C}_j \in \mathbb{R}^{(M+1)\times 1}$ *gives the element from the full trajectory* $\mathbf{X}$ *corresponding to the measurement* $\mathbf{y}_j$*. In the case* $\{t_0, ..., t_m\} \subset \{\tau_0, ..., \tau_M\}$, $\mathbf{C}_j$ *is a vector with one at position* k *satisfying* $t_j = \tau_k$*, and zeros elsewhere.*

The algorithm of course contains a burn-in period, and additional thinning, that is, not every sample l is stored.

Supplementary note 5: Incorporating several time series and knockout/knockdown experiments

Several time series experiments can be easily incorporated. For fixed $\mathbf{f}$, the probability distributions for different time series are independent. In the end, this leads to the same format of the probability distribution (7), but the trajectories are concatenated. Then $\overline{\mathbf{X}}$ contains the concatenated trajectories, except for the first point in each separate discretised trajectory, and $\underline{\mathbf{X}}$ contains all trajectories, except for the last points in each trajectory.

In a knockout experiment a particular gene is "de-activated", meaning that its expression is artificially put to zero. From an experiment where gene i has been knocked out, it is not possible to deduce anything about f_i, since the dynamics of the ith gene are artificially tampered with. Therefore these experiments are excluded from the cost functions corresponding to f_i.

In a steady state experiment, the system is allowed to evolve a long time without any excitation, so that it finally attains a steady state, where it should hold that $\mathbf{f}(\mathbf{x}_{\text{ss}}) = 0$. In the method, some noise is added to steady state measurements, and therefore, at a steady state point $\mathbf{x}_{\text{ss}}$, it is assumed that $f_i(\mathbf{x}_{\text{ss}}) = v_{i,\text{ss}}$, where $v_{i,\text{ss}} \sim \mathcal{N}(0, M_{\text{ss}})$. The incorporation of the steady state data to (7) is done by replacing $\mathbf{K}_i(\underline{\mathbf{X}})$, $\overline{\mathbf{X}}_i - \underline{\mathbf{X}}_i$, $\Delta\tau$, and $q_i\mathbf{I}$ by

$$\mathbf{K}_i([\underline{\mathbf{X}}, \mathbf{X}_{\text{ss}}]), \quad \begin{bmatrix} \overline{\mathbf{X}}_i - \underline{\mathbf{X}}_i \\ 0 \end{bmatrix}, \quad \begin{bmatrix} \Delta\tau & \\ & \mathbf{I} \end{bmatrix}, \quad \text{and} \quad \begin{bmatrix} q_i\mathbf{I} & \\ & M_{i,\text{ss}}\mathbf{I} \end{bmatrix},$$

respectively.

A steady state experiment can also be a knockout experiment. At the steady state $\mathbf{z}_i$ corresponding to knockout of gene i, it should hold that $f_j(\mathbf{z}_i) = 0$ for all j, except $j = i$, since the dynamics of gene i have been artificially tampered with.

A gene knockdown experiment is similar to a gene knockout experiment, but the genes are only repressed instead of completely inactivated, and it is taken into account in exactly the same way as a knockout experiment.

When using all of the knockout and knockdown steady state data, we assume that there is one point $\mathbf{x}_{\text{ss}}$ where $f_i(\mathbf{x}_{\text{ss}}) = 0$ for all i. This steady state value is sampled, and its prior is a normal distribution whose mean is the sample mean of all steady state measurements including the actual steady state measurement, knockout measurements, knockdown measurements, and the multifactorial data (in the DREAM4 10-gene challenge). The covariance of the prior distribution of $\mathbf{x}_{\text{ss}}$ is the sample covariance of this data, divided by the number of the steady state measurements. This corresponds to the sample covariance of the mean. We assume that at the steady state, it holds that $f_i(\mathbf{x}_{\text{ss}}) = v_{i,\text{ss}}$ where $v_{i,\text{ss}} \sim \mathcal{N}(0, M_{i,\text{ss}})$, and at the knockout and knockdown points $f_i(x_{j,\text{ko}}) = v_{i,\text{ko}}$ where $v_{i,\text{ko}} \sim \mathcal{N}(0, M_{i,\text{ko}})$. Also the covariances $M_{i,\text{ss}}$ and $M_{i,\text{ko}}$ are sampled, and they are given non-informative inverse gamma prior distributions. The incorporation of the

knockout/knockdown data to $p(\mathbf{X}|\theta)$ in (7) is done by replacing $\overline{\mathbf{X}}_i - \underline{\mathbf{X}}_i$, $\Delta\tau$, and $q_i\mathbf{I}$ by

$$\begin{bmatrix}\overline{\mathbf{X}}_i - \underline{\mathbf{X}}_i \\ 0\end{bmatrix}, \quad \begin{bmatrix}\Delta\tau & \\ & \mathbf{I}\end{bmatrix}, \quad \text{and} \quad \begin{bmatrix}q_i\mathbf{I} & & \\ & M_{i,\mathrm{ss}} & \\ & & M_{i,\mathrm{ko}}\mathbf{I}\end{bmatrix},$$

respectively, and $\mathbf{K}_i(\underline{\mathbf{X}})$ is replaced by $\mathbf{K}_i([\underline{\mathbf{X}}, \mathbf{x}_{\mathrm{ss}}, \mathbf{y}_{i,\mathrm{ko/kd}}])$ where $\mathbf{y}_{i,\mathrm{ko/kd}}$ denotes the collection of all knockout/knockdown measurements except for the ko/kd of gene i.

Supplementary note 6: Pseudo-input scheme

Gaussian process regression suffers from a very unfavourable scaling of the computational load with respect to the number of data points. This problem is further aggravated by our scheme, where the number of data points used in the GP regression is in fact the number of discretisation points in the continuous time trajectory. However, we can resort to a pseudo-input scheme, where this scaling becomes linear.

In the pseudo-input scheme [2], the underlying Gaussian process $\mathbf{f}$ is characterised through so-called pseudo-data $P := \{(\bar{\mathbf{x}}_j, \bar{\mathbf{f}}_j)\}_{j=1}^{p}$, where $\bar{\mathbf{f}}_j = \mathbf{f}(\bar{\mathbf{x}}_j)$. The number of pseudo-inputs p is specified by the user, based on the available computing power and the size of the original problem. The pseudo-inputs are not related to the inputs of the actual data, but instead they can be considered as hyperparameters, and they can be estimated by a maximum likelihood approach or they can be sampled as well. Another approach is to use only a subset of the actual input-output data (a so-called active set) in the regression [3]. We use the pseudoinput approach of [2], but the main idea then follows [3], that is, the value $\mathbf{f}(\mathbf{x})$ at a generic point $\mathbf{x}$ is approximated by $\mathbb{E}(\mathbf{f}(\mathbf{x})|P)$. When the pseudo-outputs $\bar{\mathbf{f}}_j$ are integrated out, the approximation leads to replacement of the matrices $\mathbf{K}_i(\underline{\mathbf{X}})$ in (7) by

$$\mathbf{K}_i(\underline{\mathbf{X}}) \approx \mathbf{K}_i(\underline{\mathbf{X}}, P)\mathbf{K}_i(P)^{-1}\mathbf{K}_i(\underline{\mathbf{X}}, P)^{\top},$$

where $\mathbf{K}_i(\underline{\mathbf{X}}, P) \in \mathbb{R}^{M\times p}$ is a matrix whose element (j,k) is $k_i(\mathbf{X}_{\tau_{j-1}}, \bar{\mathbf{x}}_k)$. Similarly $\mathbf{K}_i(P) \in \mathbb{R}^{p\times p}$ is a matrix whose element (j,k) is $k_i(\bar{\mathbf{x}}_j, \bar{\mathbf{x}}_k)$. The approximation used in [2] is more accurate, but its computational cost is much higher when it is not used only for regression.

With this approximation, it is possible to use the Woodbury identity and the matrix determinant lemma again to obtain for the exponent in (7)

$$\begin{aligned}&\big(\Delta\tau\mathbf{K}_i(\underline{\mathbf{X}}, P)\mathbf{K}_i(P)^{-1}\mathbf{K}_i(\underline{\mathbf{X}}, P)^{\top}\Delta\tau + q_i\Delta\tau\big)^{-1} \qquad (9)\\ &= (q_i\Delta\tau)^{-1} - \frac{1}{q_i}\mathbf{K}_i(\underline{\mathbf{X}}, P)\big(q_i\mathbf{K}_i(P) + \mathbf{K}_i(\underline{\mathbf{X}}, P)^{\top}\Delta\tau\mathbf{K}_i(\underline{\mathbf{X}}, P)\big)^{-1}\mathbf{K}_i(\underline{\mathbf{X}}, P)^{\top}.\end{aligned}$$

Here $q_i\Delta\tau$ is a diagonal matrix and the full matrix inverse is computed for a $p \times p$ matrix instead of $M \times M$. The downside is that the determinant term becomes

$$\begin{aligned}&\left|\Delta\tau\mathbf{K}_i(\underline{\mathbf{X}},P)\mathbf{K}_i(P)^{-1}\mathbf{K}_i(\underline{\mathbf{X}},P)^\top\Delta\tau + q_i\Delta\tau\right| \\ &= |\mathbf{K}_i(P)|^{-1}|q_i\Delta\tau|\left|\mathbf{K}_i(P) + \frac{1}{q_i}\mathbf{K}_i(\underline{\mathbf{X}},P)^\top\Delta\tau\mathbf{K}_i(\underline{\mathbf{X}},P)\right|\end{aligned} \quad (10)$$

where $|\mathbf{K}_i(P)|$ must be computed separately. Notice, for example, that $|\mathbf{K}_i(P)|$ tends to zero if two pseudo-inputs tend to each other, so it has an effect of pushing the pseudo-input points apart from each other. In practical implementation, a small increment $\varepsilon\mathbf{I}$ is added to the matrix $\mathbf{K}_i(P)$ to ensure numerical stability. This corresponds to assuming that the pseudo-outputs $\bar{\mathbf{f}}_j$ are corrupted by small noise (with variance $\varepsilon\mathbf{I}$). We sample the pseudoinputs using random walk sampling, using a uniform prior for the pseudoinputs in the hypercube covering the actual data.

Supplementary note 7: Crank–Nicolson sampling

In the presented algorithm, the discretised trajectory $\mathbf{X}$ is sampled using MCMC. When the discretisation is refined, the acceptation rate tends to decrease when conventional samplers are used. This can be avoided by Crank–Nicolson sampling [4, 5], if the target distribution has a density with respect to a Gaussian measure,

$$p(\mathbf{z}) = \Phi(\mathbf{z})\mathcal{N}(\mathbf{z}; \mathbf{m}, \mathbf{P}).$$

The Crank–Nicolson sampling then works as follows. Assume the current sample is $\mathbf{z}^{(l)}$. The candidate sample is $\hat{\mathbf{z}} = \mathbf{m} + \sqrt{1-\varepsilon^2}(\mathbf{z}^{(l)} - \mathbf{m}) + \varepsilon\xi$, where $\xi \sim \mathcal{N}(0, \mathbf{P})$. The new sample is then accepted with probability $\min\left\{1, \Phi(\hat{\mathbf{z}})/\Phi(\mathbf{z}^{(l)})\right\}$. The step length parameter $\varepsilon \in (0, 1)$ is chosen by the user.

Crank–Nicolson sampling plays well along with the pseudo-input scheme. The term $(q_i\Delta\tau)^{-1}$ in (9), and the term $|q_i\Delta\tau|$ in the determinant (10) correspond exactly to the Wiener measure on the discretised trajectory. Notice that also the data fit term $p(\mathbf{Y}|\mathbf{x}, \theta)$ is Gaussian. In order to get a sampler producing reasonable trajectory candidates, the Wiener measure is factorised into

$$\mathcal{W}(\mathrm{d}\mathbf{x}) = \prod_{j=1}^{m} \mathcal{N}\big(\mathbf{x}_{t_j} - \mathbf{x}_{t_{j-1}}; 0, \mathbf{Q}(t_j - t_{j-1})\big)\mathcal{B}_{(t_{j-1},t_j)}(\mathrm{d}\mathbf{x}),$$

where $\mathcal{B}_{(t_{j-1},t_j)}(\mathrm{d}\mathbf{x})$ is the Brownian bridge measure on interval (t_{j-1}, t_j), that is fixed to values $\mathbf{x}_{t_{j-1}}$ and $\mathbf{x}_{t_j}$ at the end points. Finally, the Gaussian

measure that is used in the Crank–Nicolson sampler is

$$\mathcal{N}(\mathbf{Y}|\mathbf{x},\theta)\prod_{j=1}^{m}\mathcal{B}_{(t_{j-1},t_j)}(\mathrm{d}\mathbf{x}),$$

and the factors $\prod_{j=1}^{m}\mathcal{N}\big(\mathbf{x}_{t_j}-\mathbf{x}_{t_{j-1}};0,\mathbf{Q}(t_j-t_{j-1})\big)$ are implemented in the acceptance probability.

Supplementary note 8: Prior specifications

In the experiments, the time series were scaled so that the difference of the maximal and minimal expression value for each gene was one, so that parameter priors would be consistent across dimensions. The scaling is not completely necessary, since the priors are either scale free, or are scaled accordingly if either the data is scaled or the time axis is scaled. The priors for the parameters are as follows:

- Noninformative inverse gamma prior for the process noise covariance q_i, measurement noise covariance r_i, and the steady state covariance $M_{i,\mathrm{ss}}$

$$p(q_i)\propto\frac{1}{q_i^{1.001}}\exp\left(-\frac{0.00001}{q_i}\right),$$

- Exponential priors for $a_i\geq 0$, $b_i\geq 0$, and $\beta_{i,j}$

$$p(a_i)\propto\exp\left(-\frac{a_i}{10V(\mathbf{Y}_i)}\right),\quad p(b_i)\propto\exp\left(-\frac{b_i}{5V(\mathbf{Y}_i)}\right),\quad p(H_{i,j})\propto\exp\left(-\frac{H_{i,j}}{\mathrm{ran}(\mathbf{Y}_j)}\right),$$

  where $V(\mathbf{Y}_i)$ is the variation of ith component of the trajectory per time unit (approximated from data), and $\mathrm{ran}(\mathbf{Y}_j)$ is the range of the jth trajectory:

$$V(\mathbf{Y}_i)=\frac{1}{t_m-t_0}\sum_{j=1}^{m}|[\mathbf{y}_j]_i-[\mathbf{y}_{j-1}]_i|\qquad\text{and}\qquad\mathrm{ran}(\mathbf{Y}_j)=\max_k[\mathbf{y}_k]_j-\min_k[\mathbf{y}_k]_j.$$

  Note that $\mathrm{ran}(\mathbf{Y}_j)=1$ if the time series are scaled as described above.

- Gamma prior (truncated) for γ_i

$$p(\gamma_i)\propto\gamma_i\exp\left(-\frac{\gamma_i}{5\sigma(\Delta\mathbf{Y}_i)}\right)(30-\gamma_i/\sigma(\Delta\mathbf{Y}_i))$$

  where $\sigma(\Delta\mathbf{Y}_i)$ an estimate of the variance of the derivative of the ith component of the trajectory:

$$\sigma(\Delta\mathbf{Y}_i)=\frac{1}{m}\sum_{j=1}^{m}\left(\frac{[\mathbf{y}_j]_i-[\mathbf{y}_{j-1}]_i}{t_j-t_{j-1}}\right)^2.$$

- Inverse gamma prior for the knockout measurement covariance

$$p(M_{i,\text{ko}}) \propto \frac{1}{M_{i,\text{ko}}^{N_{i,\text{ko}}/2}} \exp\left(-\frac{N_{i,\text{ko}}\sigma(\Delta\mathbf{Y}_i)}{10M_{i,\text{ko}}}\right)$$

  where $N_{i,\text{ko}}$ is the number of knockout measurements taken into account when inferring links pointing to gene i.

  Ideally also $M_{i,\text{ko}}$ should have a noninformative prior, but it was observed that this variable had a tendency to become either very small, thereby giving all weight to the knockout data and neglecting the time series data, or very large with the opposite effect. This might be due to some mismatch in the time series data and the knockout data. Nevertheless, using all data simultaneously still seemed to produce best results, but in order to achieve a good balance between both data types, the values for $M_{i,\text{ko}}$ have to be forced to a good range using an informative prior like this.

Other details of the numerical examples are presented in Supplementary table 2. The only parameter that the user has to choose is the sparsity parameter η in the topology prior $p(\mathbf{S})$. As noted in the main text, in all experiments this parameter was set to $\eta = 1/n$ where n is the dimension of the system. This prior roughly corresponds to having one incoming link for each node. This choice was not optimal — it seems that in the DREAM4 dataset the performance was better with bigger η, but with the simulated circadian clock and the IRMA data, the performance was better with smaller η. This might be due to richness of the data. In the DREAM4 data there are several time series for each network, and the majority of the network could be inferred correctly. The circadian clock network, on the other hand, was inferred from one time series, and in this case it is not possible to get the full network at once. In all experiments, the time discretisation for the Euler discretised trajectory was four times finer than the measurement discretisation, that is, three sampling points between two measurements. For the DREAM4, size 100 case the method was parallelised to three processors, each carrying out MCMC sampling independently. The number of pseudoinputs was 50 in all experiments.

Supplementary note 9: Remarks on the benchmark data examples

Remark 3. *The AUROC and AUPR values for the method CSI for the DREAM4 data are taken from [6], where the self interactions are included in the computation of these values. The self interactions are given a weight zero, and hence all methods get 10 or 100 "free" true negatives, depending on the network size. This has some increasing effect on the AUROC values for*

networks of size 10 (they report mean AUROC of 0.55 for random networks as opposed to 0.5). The effect on the 100-gene network results and on all AUPR values is negligible.

Remark 4. *In the ARNI method, the user has to choose the type and the order of basis functions. In the DREAM 10-gene case, we tried all basis function sets provided in their Matlab implementation with a variety of orders, and the best performing combinations were tried with the 100-gene case. The best performance overall in the different DREAM4 inference tasks was achieved with polynomial basis functions with degree 3. The values reported in all the results in the main text are obtained with these basis functions. In the article [7], a method for basis function selection has been introduced, but it was not implemented.*

The ARNI method considers a regression problem with input-output pairs $\left(\frac{\mathbf{y}_j+\mathbf{y}_{j+1}}{2}, \frac{\mathbf{y}_{j+1}-\mathbf{y}_j}{\Delta t}\right)$ *where* $\{\mathbf{y}_j\}$ *is the time series data. We made a small modification to the implementation by replacing the inputs by* $\mathbf{y}_j$ *which improved the method's performance.*

We could not reproduce exactly the dynGENIE3 results for the DREAM4 in silico network inference challenge data reported in [8]. We obtained similar results, but the scores for the different networks varied from the reported scores. Finally, we decided to include results from our own simulations taking into account the perturbations, whereby the results improved slightly. The inputs were incorporated by including five (or ten in the 100-gene case) additional signals to the time series, of which the jth *signal consisted of 10 ones and 11 zeros in the* j^{th} *experiment, and only zeros in other experiments.*

We used the "random forest" option with $K = n$ *in the DREAM4 experiment (as in [8]), but in other experiments we used* $K = \sqrt{n}$ *which is the default setting in the dynGENIE3 code.*

For GRNTE, all time series were used at once as replicates with the DREAM4 data. However, the IRMA data is not evenly sampled, and sampling times differ for different time series. Therefore the method was applied separately on each time series, and an average network was used as the end result.

Remark 5. *In the analysis of all the results, we have ignored self-regulation as was done in the DREAM4 challenge. Therefore, in the IRMA network, the maximum number of possible links is 20, whereas in references [9, 10] the self-regulation is somehow taken into account (details are not given), and the number of possible links is 25. Moreover, it seems that in [10] one link (Gal4* $\rightarrow$ *Swi5) has been omitted from the ground truth. With these criteria, using the ground truth network from [11], shown in Supplementary figure 1, the ELM-GRNNminer had 6 true positives (out of eight links in the ground truth network) and 3 false positives (out of* $20 - 8 = 12$ *missing links in the ground truth), and TD-ARACNE had 5 true positives and 2 false positives. These numbers were used to plot the predictions in Figure 2 of the main text.*

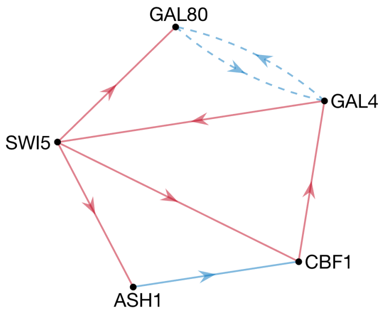

Supplementary figure 1: The ground truth IRMA network (from [11]). Red arrows denote positive regulation, and blue arrows denote inhibitory regulation. Dashed arrows mean protein-protein interaction.

Supplementary table 2: Simulation details on the benchmark examples. In DREAM4 size 100, three independent chains were run in parallel. The total number of sampling rounds is the burn-in length added to the number of samples multiplied by the thinning factor. The computational times are for inferring one network. They are obtained with a Macbook pro, 2.4 GHz Intel Core i7, except for the DREAM4, size 100 and NAM cases, which are with Dell, 2.5 GHz Intel Xeon E5-2680 v3.

Experiment	η	Burn-in	Nr. samples	thinning factor	computation time (min)
DREAM4, size 10	1/10	3000	10000	10	31
DREAM4, size 100	1/100	1500	3×3000	10	3×188
Circadian clock	1/7	3000	6000	10	(1h/2h/4h) 6/5/4
IRMA	1/5	3000	10000	10	(avg./full) 7/18
NAM	1/994	1000	20×750	10	20×1135

Supplementary table 3: Computation times for all methods in the *Arabidopsis thaliana* example with 4h sampling rate. Results are obtained by different computers with similar capacity.

Method	computation time (s)
BINGO	217
dynGENIE3	0.7
ARNI	1.0
iCheMA	1999
GRNTE	30.0

Supplementary table 4: AUROC/AUPR values for the DREAM4 *in silico* 10-gene (above) and 100-gene (below) network inference challenge data, using either all data or only time series data. The values for PNFL and the 100-gene challenge winner are taken from [12], for dynGENIE3*MCZ from [8, Suppl. information], and for CSI from [6, Table 1] (see Supplementary note 9 for a remark on the AUROC values). The MCZ method we implemented ourselves, and for the dynGENIE3, iCheMA and ARNI results, the codes provided by the authors of [8], [13] and [7], respectively, were used.

	Data	Method	Network 1	Network 2	Network 3	Network 4	Network 5	Average
Size 10	TS	BINGO	.882 / .829	.790 / .704	.782 / .567	.933 / .835	.954 / .882	.868 / .763
		CSI	(.72) / .64	(.75) / .54	(.67) / .45	(.83) / .67	(.90) / .78	(.77) / .62
		dynGENIE3	.743 / .551	.715 / .463	.765 / .543	.802 / .706	.923 / .790	.790 / .611
		iCheMA	.576 / .401	.733 / .445	.770 / .464	.563 / .273	.677 / .357	.664 / .388
		ARNI	.835 / .682	.779 / .626	.665 / .280	.768 / .387	.873 / .355	.784 / .466
		GRNTE	.728 / .481	.682 / .529	.729 / .390	.774 / .532	.731 / .611	.729 / .509
	All	BINGO	.941 / .854	.877 / .779	.936 / .787	.957 / .862	.928 / .830	.928 / .822
		PNFL	.972 / .916	.841 / .547	.990 / .968	.954 / .852	.928 / .761	.937 / .809
		dynGENIE3*MCZ	NA / .82	NA / .60	NA / .80	NA / .77	NA / .59	NA / .72
		BINGO*MCZ	.972 / .865	.854 / .703	.893 / .738	.966 / .909	.938 / .846	.925 / .812
	KO+KD	MCZ	.941 / .813	.728 / .306	.832 / .662	.923 / .713	.717 / .391	.828 / .577

	Data	Method	Network 1	Network 2	Network 3	Network 4	Network 5	Average
Size 100	TS	BINGO	.816 / .447	.741 / .296	.781 / .345	.787 / .407	.807 / .438	.786 / .386
		dynGENIE3	.789 / .276	.700 / .175	.770 / .271	.736 / .248	.766 / .214	.752 / .237
		CSI	.71 / .25	.67 / .17	.71 / .25	.74 / .24	.73 / .26	.71 / .23
		ARNI	.726 / .159	.641 / .098	.689 / .109	.683 / .129	.696 / .116	.687 / .122
		GRNTE	.746 / .230	.680 / .147	.702 / .201	.707 / .195	.740 / .169	.715 / .188
	TS+KO	BINGO	.857 / .485	.750 / .322	.796 / .404	.819 / .435	.828 / .456	.810 / .420
	All	BINGO	.823 / .404	.725 / .243	.770 / .299	.777 / .325	.788 / .296	.777 / .313
		DREAM4 winner	.914 / .536	.801 / .377	.833 / .390	.842 / .349	.759 / .213	.830 / .373
		dynGENIE3*MCZ	NA / .60	NA / .43	NA / .47	NA / .52	NA / .37	NA / .48
		BINGO*MCZ	.911 / .588	.813 / .400	.870 / .447	.856 / .510	.850 / .464	.860 / .482

Supplementary table 5: Means and standard deviations of AUROC/AUPR values for the simulated circadian clock data with ten replicates.

	Method	1h sampling	2h sampling	4h sampling
Low noise	BINGO	$.674 \pm .052/.658 \pm .069$	$.653 \pm .061/.645 \pm .060$	$.664 \pm .060/.632 \pm .068$
	dynGENIE3	$.659 \pm .025/.500 \pm .054$	$.671 \pm .039/.504 \pm .048$	$.651 \pm .043/.515 \pm .060$
	iCheMA	$.606 \pm .061/.463 \pm .068$	$.503 \pm .096/.356 \pm .059$	$.542 \pm .120/.286 \pm .045$
	ARNI	$.590 \pm .074/.444 \pm .065$	$.600 \pm .069/.441 \pm .058$	$.503 \pm .055/.395 \pm .062$
	GRNTE	$.361 \pm .035/.367 \pm .032$	$.443 \pm .059/.324 \pm .028$	$.549 \pm .062/.396 \pm .047$

	Method	1h sampling	2h sampling	4h sampling
High noise	BINGO	$.821 \pm .040/.824 \pm .037$	$.830 \pm .032/.818 \pm .040$	$.808 \pm .035/.780 \pm .038$
	dynGENIE3	$.641 \pm .027/.536 \pm .027$	$.644 \pm .039/.546 \pm .048$	$.608 \pm .101/.512 \pm .091$
	iCheMA	$.693 \pm .054/.584 \pm .045$	$.700 \pm .061/.610 \pm .055$	$.475 \pm .098/.269 \pm .041$
	ARNI	$.666 \pm .051/.591 \pm .071$	$.643 \pm .074/.516 \pm .080$	$.588 \pm .055/.479 \pm .094$
	GRNTE	$.463 \pm .046/.362 \pm .044$	$.517 \pm .051/.403 \pm .047$	$.590 \pm .041/.479 \pm .061$

Supplementary table 6: AUROC/AUPR values for the IRMA dataset using either the two averaged time series or all nine time series.

Method	Avg. data	Full data
BINGO	.833/.800	.823/.765
dynGENIE3	.635/.586	.656/.521
iCheMA	.490/.373	.594/.515
ARNI	.521/.395	.552/.404
GRNTE	.417/.436	.417/.450

Supplementary references

[1] Wang, J. *Gaussian Process Dynamical Models for Human Motion.* M.Sc. Thesis, (University of Toronto, 2005).

[2] Snelson, E. & Ghahramani, Z. Sparse Gaussian processes using pseudo-inputs. In *Adv. Neural Inf. Process. Syst.*, 1257–1264 (2006).

[3] Seeger, M., Williams, C. & Lawrence, N. Fast forward selection to speed up sparse Gaussian process regression. *Artificial Intelligence and Statistics* **9** (2003).

[4] Beskos, A., Roberts, G., Stuart, A. & Voss, J. MCMC methods for diffusion bridges. *Stoch. Dyn.* **8**, 319–350 (2008).

[5] Cotter, S. L., Roberts, G. O., Stuart, A. M. & White, D. MCMC methods for functions: modifying old algorithms to make them faster. *Stat. Sci.* **28**, 424–446 (2013).

[6] Penfold, C. A. & Wild, D. L. How to infer gene networks from expression profiles. *Interface Focus* **1**, 857–870 (2011).

[7] Casadiego, J., Nitzan, M., Hallerberg, S. & Timme, M. Model-free inference of direct network interactions from nonlinear collective dynamics. *Nat. Commun.* **8**, 2192 (2017).

[8] Huynh-Thu, V. A. & Geurts, P. dynGENIE3: dynamical GENIE3 for the inference of gene networks from time series expression data. *Sci. Rep.* **8**, 3384 (2018).

[9] Zoppoli, P., Morganella, S. & Ceccarelli, M. TimeDelay-ARACNE: Reverse engineering of gene networks from time-course data by an information theoretic approach. *BMC Bioinformatics* **11**, 154 (2010).

[10] Rubiolo, M., Milone, D. & Stegmayer, G. Extreme learning machines for reverse engineering of gene regulatory networks from expression time series. *Bioinformatics* **34**, 1253–1260 (2017).

[11] Cantone, I., Marucci, L., Iorio, F., Ricci, M., Belcastro, V., Bansal, M., Santini, S., di Bernardo, M., di Bernardo, D. & Cosma, M. A yeast synthetic network for in vivo assessment of reverse-engineering and modeling approaches. *Cell* **137**, 172–181 (2009).

[12] Sage Bionetworks. DREAM4 - in silico network challenge. `https://www.synapse.org/#!Synapse:syn3049712/wiki/`. Accessed: 25.05.2018.

[13] Aderhold, A., Husmeier, D. & Grzegorczyk, M. Approximate Bayesian inference in semi-mechanistic models. *Stat. Comput.* **27**, 1003–1040 (2017).
