# Supplementary material for: Gene regulatory network inference from sparsely sampled noisy data
Source: Nat Commun. 2020 Jul 13;11:3493. doi: 10.1038/s41467-020-17217-1 (PMC7359369; doi:10.1038/s41467-020-17217-1)
Supplement: Supplementary file 3 — Reporting Summary [file 41467_2020_17217_MOESM3_ESM.pdf]

nature research

Corresponding author(s): Jorge Goncalves
Last updated by author(s): Jun 2, 2020

Reporting Summary

Nature Research wishes to improve the reproducibility of the work that we publish. This form provides structure for consistency and transparency in reporting. For further information on Nature Research policies, see Authors & Referees and the Editorial Policy Checklist.

Statistics

For all statistical analyses, confirm that the following items are present in the figure legend, table legend, main text, or Methods section.

n/a	Confirmed	
☒	☐	The exact sample size (*n*) for each experimental group/condition, given as a discrete number and unit of measurement
☒	☐	A statement on whether measurements were taken from distinct samples or whether the same sample was measured repeatedly
☒	☐	The statistical test(s) used AND whether they are one- or two-sided<br>*Only common tests should be described solely by name; describe more complex techniques in the Methods section.*
☒	☐	A description of all covariates tested
☒	☐	A description of any assumptions or corrections, such as tests of normality and adjustment for multiple comparisons
☒	☐	A full description of the statistical parameters including central tendency (e.g. means) or other basic estimates (e.g. regression coefficient) AND variation (e.g. standard deviation) or associated estimates of uncertainty (e.g. confidence intervals)
☒	☐	For null hypothesis testing, the test statistic (e.g. *F*, *t*, *r*) with confidence intervals, effect sizes, degrees of freedom and *P* value noted<br>*Give P values as exact values whenever suitable.*
☐	☒	For Bayesian analysis, information on the choice of priors and Markov chain Monte Carlo settings
☒	☐	For hierarchical and complex designs, identification of the appropriate level for tests and full reporting of outcomes
☒	☐	Estimates of effect sizes (e.g. Cohen's *d*, Pearson's *r*), indicating how they were calculated

Our web collection on statistics for biologists contains articles on many of the points above.

Software and code

Policy information about availability of computer code

Data collection	No data collection was performed as part of the study
Data analysis	The method implementation is done using Matlab, which is also used for generating all plots appearing in the manuscript.

For manuscripts utilizing custom algorithms or software that are central to the research but not yet described in published literature, software must be made available to editors/reviewers. We strongly encourage code deposition in a community repository (e.g. GitHub). See the Nature Research guidelines for submitting code & software for further information.

Data

Policy information about availability of data

All manuscripts must include a data availability statement. This statement should provide the following information, where applicable:
- Accession codes, unique identifiers, or web links for publicly available datasets
- A list of figures that have associated raw data
- A description of any restrictions on data availability

We use four different datasets in the article:

1. DREAM4 in silico network inference challenge: available at https://www.synapse.org/#!Synapse:syn3049712/wiki/74630

2. Simulated data from Arabidopsis thaliana circadian clock: available as example data along with the method’s code at github.com/AtteAalto/BINGO

3. In vivo dataset IRMA: available as supplemental data for the article I. Cantone et al. “A yeast synthetic network for in vivo assessment of reverse-engineering and modeling approaches”, Cell 137:172-181, doi: https://doi.org/10.1016/j.cell.2009.01.055

4. Data from Arabidopsis thaliana circadian clock is related to publication L. Mombaerts et al. “Dynamical differential expression (DyDE) reveals the period control

mechanisms of the Arabidopsis circadian oscillator", PLoS Computational Biology 15(1):e1006674, 2019. The data is deposited at http://www.ncbi.nlm.nih.gov/geo/query/acc.cgi?acc=GSE19271

Field-specific reporting

Please select the one below that is the best fit for your research. If you are not sure, read the appropriate sections before making your selection.

☒ Life sciences ☐ Behavioural & social sciences ☐ Ecological, evolutionary & environmental sciences

For a reference copy of the document with all sections, see nature.com/documents/nr-reporting-summary-flat.pdf

Life sciences study design

All studies must disclose on these points even when the disclosure is negative.

Sample size	No data was collected as part of the study
Data exclusions	No data was excluded
Replication	No data was collected as part of the study. Only readily available data or simulated data is used. Results can be replicated by applying the method to the data. Both are available.
Randomization	Not applicable
Blinding	Not relevant. Main result of the study is the developed method, and results in the article are related to benchmarking the method.

Reporting for specific materials, systems and methods

We require information from authors about some types of materials, experimental systems and methods used in many studies. Here, indicate whether each material, system or method listed is relevant to your study. If you are not sure if a list item applies to your research, read the appropriate section before selecting a response.

Materials & experimental systems

n/a	Involved in the study	
☒	☐	Antibodies
☒	☐	Eukaryotic cell lines
☒	☐	Palaeontology
☒	☐	Animals and other organisms
☒	☐	Human research participants
☒	☐	Clinical data

Methods

n/a	Involved in the study	
☒	☐	ChIP-seq
☒	☐	Flow cytometry
☒	☐	MRI-based neuroimaging
